# The Use of Public Health Indicators to Assess Individual Happiness in Post-Disaster Recovery

**DOI:** 10.3390/ijerph16214101

**Published:** 2019-10-24

**Authors:** Yingying Sun, Tingting Yan

**Affiliations:** Institute for Disaster Management and Reconstruction, Sichuan University, Chengdu 610200, China; sunying@scu.edu.cn

**Keywords:** happiness, nuclear radiation, post-disaster recovery, Fukushima

## Abstract

Purpose: Very few studies have examined the influential factors of survivors’ feelings of happiness in the context of nuclear accidents. This paper aims to fill this gap with reference to the recovery process in Fukushima City following the 2011 Tohoku Earthquake Tsunami in Japan. Methods: Open access data were sourced from the 2015 Social Survey on Living and Disaster Recovery (SSLDR) (N = 1439) of Fukushima citizens. Pearson’s Chi-square Test and the t-test were employed to examine gender differences with regard to happiness and exploratory variables. Following this, a multiple linear regression analysis was conducted to investigate the determinants of happiness. Results: The results showed that, compared to females, male respondents were unhappier and reported more property loss and less neighborhood connectedness. Individuals’ mental and physical health and neighborhood connectedness were found to be significantly correlated with their happiness. However, the disaster-related variables of people’s evaluation of recovery achievement, concerns around the health impacts of radiation, property loss in the disaster, and experiences of casualty, had no effects on happiness. Conclusion: These findings indicate that policies and countermeasures dealing with disaster recovery over the long term should continuously focus on health issues and social relationships.

## 1. Introduction

Diverse hazards, influenced by complexities in the environment and social constructs, have resulted in population death and adverse health impacts [1,2,3]. Disaster studies have widely reported on the impact of disaster-related damage to physical and mental health, social relationships, and community development [2,4,5]. As a result of these hazardous damages, designing effective recovery policies for the short- and long-term have been recognized as key strategies for public health promotion [6,7,8].

The 2011 Tohoku Earthquake Tsunami, and the following Fukushima Daiichi nuclear accident, caused nearly 20,000 deaths, environmental and radioactive contamination, and severe public health impacts [1,9]. As a result of the nuclear accident, nearly 165,000 people immediately evacuated to other locations [10]. Since the occurrence of the nuclear accident, safety concerns over the effects of nuclear radiation on human beings, agriculture and food, and nuclear energy have become serious issues in Japanese society. These concerns have been reported to reduce the public’s well-being, including individuals’ happiness [1,11].

Happiness is derived from a representative question about how happy people are with their lives [1,12]. The conception is seen as encompassing the possession of resources [13,14], the satisfaction of needs, wants and desires [15], participation in self-actualizing activities [16], and comparisons with others and past experience [17]. Scholars acknowledge that, although the concept of well-being reflects a more stable state of being well, satisfied and contented than that of happiness, research on both of these concepts has gradually evolved in two directions based on the philosophical perspectives of hedonism and eudemonia [12,18,19]. In this sense, the current study considers happiness to be one aspect of well-being, and uses happiness as an indicator by which to measure the general status of survivors’ well-being in the context of post-disaster recovery.

A large volume of empirical research has examined the individual relationships between happiness and various demographic, sociological, psychological and behavioral characteristics [17,20,21,22]. A few recent studies even created the new term of “community happiness” to describe sustainable well-being in a broad range of social, economic, environmental and urban governance contexts, in order to capture individuals’ subjective perceptions of their experience of communities and the impact of development [23,24]. Research shows that positive emotions (like happiness) make people more resilient to disaster, with the capacity to bounce back in terms of their physiology and mood more quickly than people with negative emotions after a disaster has affected their surrounding environment [25]. Evidence following the Fukushima accident demonstrated that psychological distress exerted a longer-term impact on happiness than cancer mortality caused by actual radiation exposure [2], although there was no actual cancer mortality observed or anticipated to be observed due to radiation exposure in Fukushima Prefecture. Regarding the literature on the impacts of nuclear accidents on the public’s well-being, the first study might be Kasl et al.’s work on examining the impact of the Three Mile Island Accident on the well-being of nuclear workers [3]. Since then, detailed studies on the impacts of the Chernobyl accident on individuals’ well-being have been carried out [26,27] and, recently, several empirical studies on the impact of the Fukushima accident have been conducted [1,28]. Nevertheless, much less is known about the determinants of survivors’ happiness, or about the correlation between happiness and public health and how this interacts with individual daily lives in the context of disaster recovery. Furthermore, little is known about the impact of this recovery on individuals’ happiness.

To study these research gaps, the current study takes happiness as an indicator by which to measure the general status of survivors’ well-being in Fukushima City four years after the Tohoku Earthquake Tsunami. Based on the literature review, the following seven testable hypotheses are proposed:

**H1:**  *Good physical health has a positive impact on happiness.*

**H2:**  *Good mental health has a positive impact on happiness.*

**H3:**  *Individuals with more concerns as to how radiation may impact their health feel a lower degree of happiness than those with fewer concerns.*

**H4:**  *Experiencing casualty has a negative impact on happiness.*

**H5:**  *Experiencing property loss has a negative impact on happiness.*

**H6:**  *The better a community recovers from a disaster, the higher the degree of happiness that local people will feel.*

**H7:**  *Frequent neighborhood connectedness has a positive impact on happiness.*

The purpose of this study is to investigate the determinants of survivors’ happiness with reference to the recovery process in Fukushima City following the Tohoku Earthquake Tsunami and to provide empirical evidence for designing adequate policies and countermeasures to enhance survivors’ happiness in a post-disaster recovery context.

## 2. Materials and Methods

### 2.1. Data Collection

Open access data were sourced from the 2015 Social Survey on Living and Disaster Recovery (SSLDR). The SSLDR datasets were provided by the Center for Social Research and Data Archives, Institute of Social Science, at the University of Tokyo. The SSLDR datasets include a series of surveys conducted after the Tohoku Earthquake Tsunami. The first survey was conducted in Sendai City in 2011. The survey items were then reevaluated and modified according to previous survey results. The survey targets were adults of at least 20 years of age. The SSLDR series questionnaires were designed following the ethical standards of “Rikkyo University Standards for Life Science Research Ethics and Safety”. There was not any approved ethics number for the SSLDR.

The 2015 SSLDR was collected in Fukushima City from 4 June to 31 July 2015. A random sampling method of residential areas was applied to select the survey samples. Based on population distribution, 70 survey locations were selected. The survey headquarters randomly designated one household as the starting point on a map. Then, investigators visited households located on the map and chose one out of every five households to be surveyed in that location, with 30 households selected in each location. Investigators visited each household to choose a person to answer the questionnaires. The respondent selection criteria were such that if there were at least two persons (≥20 years old) in one household, the person whose next birthday would the first to be celebrated following the survey request would be the one to answer. Questionnaires were directly distributed to each household, and completed questionnaires were collected by house visits or were returned by post. A total of 2100 households were selected, out of which respondents from 1452 households answered and returned the questionnaires. The response rate reached 69.1% [29]. Thirteen cases with missing values for the variable of happiness were disregarded in the analysis, with a total of 1439 responses thus used.

### 2.2. Measures

This section describes the variables used in the analysis and their measurements. The original questionnaires and measured values of each variable in the 2015 SSLDR are provided in Appendix A.

#### 2.2.1. Dependent Variable

The 2015 SSLDR used one questionnaire item (“In general, do you feel happy?”) to measure individuals’ happiness. This variable was measured by four Likert scales [30] (“happy” to “unhappy”), representing the decreasing degree of the indicator.

#### 2.2.2. Explanatory Variables

##### Mental Health

The 2015 SSLDR used the Japanese version of the K6 questionnaire format to measure individuals’ level of mental health. The effectiveness of the Japanese version of the K6 questionnaire was confirmed by Furukawa et al., with the cutoff value for this K6 questionnaire set at nine [31]. The question items are as follows: (1) I feel mentally burned out, (2) I do not feel motivated to work or undertake household chores, (3) I feel restless, (4) I cannot sleep well, (5) I feel depressed. No matter what happens, I feel sick, (6) I feel too exhausted to do anything. The answers to these six questions were measured by four-point Likert scales (“agree” to “disagree”). The Cronbach’s alpha (a function of the number of items in a test, the average covariance between item-pairs, and the variance of the total score) [30] for the six items pertaining to mental health was 0.87. The sum of the six items’ scores was used to represent the variable of mental health. The higher the score, the better the mental health. 

##### Physical Health

Physical health was measured by asking the following question: “Overall, how would you describe the current condition of your health?” This question was taken from the questionnaire of the Ninth International Comparative Survey on Values and Behavioral Patterns of the 2012 Japan General Social Survey. The answers were given according to five-point Likert scales (“good” to “bad”), representing the decreasing degree of the indicator. 

##### Radiation Related Concerns

Individuals’ concerns relating to the impact of nuclear radiation on health were measured by the following question: “do you agree that negative health effects caused by nuclear radiation due to the Fukushima accident will emerge in the future?” The answers were measured by four-point Likert scales (“agree” to “disagree”). The higher the score, the lower the concern over radiation.

##### Experience of Casualty

The 2015 SSLDR used a total of five items to measure individual experiences of casualties in the Tohoku Earthquake Tsunami, as follows: (1) I was injured, (2) family or relatives were injured, (3) family or relatives died, (4) friends or acquaintances were injured, (5) friends or acquaintances died. The answers were provided in ‘yes’ or ‘no’ format, generating five dummy variables related to individual experiences of casualties for the purposes of the current analysis.

##### Property Loss

Property loss was measured by asking the question: “how much damage was there to your property, such as houses or household goods?” Respondents were asked to give a specific amount of money in Japanese Yen (JPY).

##### Feelings of Recovery Achievement

Individuals’ feelings of recovery achievement were measured by asking the question: “four years after the Tohoku Earthquake Tsunami and Fukushima accident, how far do you think the goals of recovery in Fukushima Prefecture have been achieved?” This variable was measured by four-point Likert scales (“very good” to “very bad”), representing the decreasing degree of the indicator.

##### Neighborhood Connectedness

Neighborhood connectedness was measured by asking the following two questions: (1) “How often do you talk with your neighbors?” (2) “How often do you tell your neighbors about your troubles or vice versa?” Both questions were measured by four-point Likert scales (“often” to “never”), representing the decreasing degree of each indicator. The sum of the answer scores to these two questions represented the value of the variable of neighborhood connectedness.

#### 2.2.3. Control Variables

Age was applied as a continuous variable, ranging from 20 to 93 years. Gender was a dichotomic variable. Marital status was measured according to three categories: married, single, and divorced/widowed. Education was measured by seven categories and was recoded into three groups: vocational school or below (elementary school, middle school, high school, vocational school), college or above (college, undergraduate school and above), and “other schooling” (e.g., old-style private schools, such as those in operation prior to World War II). With regard to family annual income (FAI), the 2015 SSLDR used a total of 15 measurement categories (see Appendix A). As the average FAI of Japan is approximately 5.5 million JPY in 2015, FAI was recoded into five levels: under 1.5, 1.5–4.5, 4.5–6.5, 6.5–10, and over 10 million JPY.

### 2.3. Analysis Methods

First, prior to conducting the statistical analysis, missing data were analyzed within the 1439 responses and seen in 731 data values (3.18%) out of 23,024 total data values. The result of Little’s Missing Completely at Random (MCAR) test [30] showed that the missing data were not missing completely at random (*p* < 0.00). Thus, the method of Multiple Imputations was used to replace those missing data [32,33]. Finally, an original dataset was obtained without missing data to the effect that, among the selected variables, the minimum number of respondents was 1232. Five imputed datasets with the missing data replaced thus contained 1439 respondents.

Then, with regard to descriptive statistics, Pearson’s Chi-square Test and t-test were applied to assess the differences between male and female responses. For the regression analysis, a multiple linear regression analysis was applied in order to examine the determinants of happiness. The stepwise method was used to eliminate variables until the p-value of all variables in the regression became significant (*p* < 0.05). The multiple linear regression analysis was conducted for the original dataset (N = 1232), and then the same was done for the imputed datasets (N = 1439), in order to check for concurrence between these two types of datasets. All of these analyses were performed using IBM SPSS 23 (IBM Corporation, Armonk, 2014). 

## 3. Results

### 3.1. Response Characteristics

The original dataset was used to conduct the descriptive statistical analysis by using gender as the stratum. These results are shown in Table 1. There were more male respondents than females. Compared to females, male respondents were found to be older and unhappier, reported more property loss, and had less neighborhood connectedness. With regard to neighborhood connectedness, both male and female respondents reported having infrequent contact with their neighbors. The mean value of reported property loss emerged as significantly different between males and females. Furthermore, significant differences were found between male and female respondents in terms of marriage, education, and FAI.

With regard to the variables of feelings of recovery achievement, concerns over the health impacts of radiation, physical health, mental health and experiences of casualty, no significant differences emerged between male and female respondents. Specifically, respondents’ feelings of the recovery achievement in the Fukushima Prefecture was quite negative. A similar negative report also emerged in relation to respondents’ physical health. Overall, respondents reported their mental health to have improved. Concerns about the impacts of radiation on health seemed to be neutral among both males and females. In addition, the pooled results of the imputed datasets (N = 1439) showed the same attributes as those of the original dataset (Appendix B).

### 3.2. Multiple Linear Regression Analysis

The minimum samples of the original dataset (N = 1232) were taken in order to conduct a multiple linear regression analysis of happiness and the independent variables. These results are shown in Table 2. Happiness was found to be significantly positively correlated with physical health and neighborhood connectedness. The negative correlations between mental health and happiness indicate that people who reported having good mental health tended to be happy. Accordingly, young people also tended to be happy. Compared to females, Table 2 shows that males tend to be unhappy. Compared to married people, those who reported being single and divorced/widowed tended to be more unhappy. Compared to people who had received education from “other schooling”, respondents who had completed a college-level education or above tended to be happier. Compared to people whose FAI was over 10 million JPY, respondents whose FAI fell between 6.5–10 million JPY tended to be happier. However, the disaster-related variables of feelings of recovery achievement, concerns over radiation, property loss, and experiences of casualty were not significantly correlated with happiness.

With regard to the imputed datasets (N = 1439), the multiple linear regression analysis results showed almost the same tendencies as those of the original dataset. Nonetheless, the variable of having an FAI of 6.5–10 million JPY was not significantly correlated with happiness (Appendix C).

## 4. Discussion

This study examines the influential factors of happiness in the process of four-year post-disaster recovery. The results showed that females reported higher levels of happiness than males. While this result is in line with some previous studies [34], it stands in contrast to other studies related to post-disaster recovery [27,35,36]. For example, following their review of a large volume of research on well-being, Diener, Lucas, and Oishi [19] state that sex differences in well-being are not universal, and depend on different societies’ cultural values and conditions. The current results demonstrate a difference between the sexes with regard to happiness. This may be explained by the fact that the female respondents were younger than the male ones. That is to say, arguably, the female respondents’ youth may have meant they had more energy with which to handle the chaos or new policies potentially related to household reconstruction, compensation, or employment during the post-disaster recovery process. Following the Tohoku Earthquake Tsunami, large areas of communities were flooded away. Accordingly, businesses, schools, and even whole communities had to be reconstructed. This new environment thus required people to invest more energy into daily life activities than in the pre-disaster period. Hence, as age is a core factor in determining people’s physical activity, females who were younger could be seen as adapting more easily to this new context and thus feeling happier than males. This difference between the sexes with regard to the relationship between age and happiness in the context of post-disaster recovery is a new finding as no previous studies in the field, such as those of Ardalan et al. [35] and Wu et al. [36], have engaged with this aspect.

Regarding property loss in the Tohoku Earthquake Tsunami, the present results indicate that males were inclined to report more property loss than females. This result might be explained by the fact that men are, generally speaking, the main breadwinners in Japanese households. Moreover, as the mean age of the male respondents was high, some of them may have had difficulties participating in the post-disaster recovery process. Thus, they may have reported a greater amount of property loss in order to obtain a higher subsidy to help them reconstruct their homes or to buy new household goods. Another explanation could be that people tend to over-estimate the value of the goods they paid for.

Regarding respondents’ feelings of the actual achievement of recovery in Fukushima Prefecture, the results showed an extremely negative attitude towards this. The reasons here might be that, first, such an achievement requires a huge financial budget for disaster recovery resources and, therefore, a long period of time in which to realize the implementation of this budget. For instance, according to the Reconstruction Agency [37], there were about 16.9 trillion JPY in direct financial damages due to the Tohoku Earthquake Tsunami, with over 23 trillion JPY required for reconstruction over the next decade. This long period of time needed by the recovery process may have incurred an inconvenience for Fukushima citizens who wanted to return to their normal lives as soon as possible. This unconformity between local needs and the actual progress of recovery may thus have generated negative attitudes towards recovery achievement. Second, objectively speaking, the completion rate of this reconstruction has, indeed, been comparatively low. In terms of the current reconstruction situation, according to Miyagi Prefecture’s report of 30 April 2019, the coastal facilities have achieved a 50% completion rate and channel facilities a 20% completion rate, with reconstruction land for households only just having been elevated to a safe height in order to mitigate future tsunami risks [38].

With regard to the seven hypotheses posited in the current paper, only three were supported by the results. Specifically, hypothesis H1 (that good physical health has a positive impact on happiness), H2 (that good mental health has a positive impact on happiness), and H7 (that frequent neighborhood connectedness has a positive impact on happiness), were clearly supported by the multiple linear regression results. These results are in line with previous literature that has explored the influencing factors on happiness [14,21,39].

Regarding the findings around mental and physical health, the explanations for this could include the notion that happiness involves positive emotions [12], and that people who experience positive emotions are thus more resilient than those who experience negative emotions, in terms of their physiology and mood after a disaster or stressor [25,40]. Moreover, as previously mentioned, in the process of recovery following the Tohoku Earthquake Tsunami, people had to invest much energy into reconstructing businesses, schools, and even whole communities, all of which require good physical health. Previous studies on post-disaster recovery have also revealed decreased dependency in daily activities to be associated with high levels of happiness [35].

Regarding neighborhood connectedness, its positive impacts on happiness have been verified by numerous studies [12,18,35,41]. Specifically, in studies relating to post-disaster recovery, scholars have reported that people who continued to have low levels of happiness during the post-disaster recovery period were more likely to be living on their own, or had received little social support from the community [35,41]. Frequent neighborhood connectedness could alleviate psychological stress, and help individuals to obtain effective social support and quickly adapt to a new environment after a disaster [42,43], which may, in turn, make them feel happy. In addition, the current results showed that compared to males, females tended to have more frequent contact with neighbors. This result is quite easy to understand because, even in current Japanese society, women (especially housewives) are the main connectors between the family and wider community. Generally speaking, the gender role division is such that women are expected to be responsible for arranging various community events, such as attending parents’ meetings at schools, preparing food for the elderly in the community, or arranging schedules for local festivities. Conversely, men’s central responsibility is that of working hard and earning salaries. Due to the large-scale evacuations or relocations following the Tohoku Earthquake Tsunami, the damage to pre-disaster neighborhood connectedness could thus have reduced the amount of social support available and exerted psychological distress. In light of this, and according to the gender role division described above, men who had to focus on work had fewer opportunities to build new connections than women. In light of these social contexts, Japanese women’s more frequent neighborhood connections may, therefore, be what contributed to their higher happiness than men’s in the present study.

The other four hypotheses—namely, H3 (that individuals with greater concerns about radiation affecting health feel less happiness than those with fewer concerns), H4 (that experiencing a form of casualty has a negative impact on happiness), H5 (that experiencing property loss has a negative impact on happiness), and H6 (that the better a community recovers from a disaster, the higher the level of happiness local people feel)—could not be verified by the results of this research. Regarding the results pertaining to H3 and H4, these are in line with previous studies [41,44] and can be explained by the fact that people may already have recovered from psychological distress given that four years had passed since the disaster. 

The results with regard to H5 are not consistent with previous studies. Given the common wisdom that possessing certain resources may afford people pleasure, such as the universal doctrine positing that ‘money buys happiness’ [4,45], a loss of property in the context of a disaster could be seen as reducing happiness. However, according to the current results, compared to FAI, the average property loss was quite low. Hence, four years after the Tohoku Earthquake Tsunami, people may have earned more than enough to compensate for their property loss. Alternatively, this property loss may have been compensated for by insurance companies, or by the local or central government.

Regarding the achievement of recovery, the results showed no correlations with happiness. As daily life is built on physical and institutional systems [46,47], to some extent, the degree of recovery achievement can be seen as impacting on happiness. Nevertheless, happiness is typically seen as consisting of the possession of resources, the satisfaction of needs and desires, and participation in self-actualizing activities, all of which relate to self-fulfillment [13,15,16]. Conversely, the variable of individuals’ evaluation of recovery achievement was a judgment regarding the work of other people (i.e., government, society, community), which does not have a direct relationship with an individual’s own efforts and achievements. Making such a judgment about others’ work may thus be the reason why recovery achievement, in the context of this research, was not found directly to impact on happiness.

## 5. Conclusions

The findings of the current study show that certain factors (e.g., mental and physical health, neighborhood connectedness), showed significant correlations with happiness, as largely verified by previous studies. Conversely, disaster-related variables (e.g., feelings of recovery achievement, concerns about radiation, property loss, the experience of casualty) showed no significant correlations with happiness. The latter results imply that policies and countermeasures dealing with disaster recovery over the long term should focus more on health issues and social relationships. For example, as well as establishing projects for household reconstruction, the following are also important: providing employment support and subsidy distributions, designing medical services programs, and community sustainable development (e.g., community festivals, community center activities). Again, the current results verify the common knowledge that the direct impacts caused by a disaster can be alleviated as time goes by. People who are living in or around disaster-affected areas eventually adapt to the newly changed environment and continue to go about their daily lives.

## 6. Limitations

The present study has a number of limitations. Firstly, the data did not enable the examination of specific concerns over radiation impacts. Concerns over the impacts of radiation on food, seafood, and pollution to the ecosystem, and with regard to energy policies on nuclear power plants, are ongoing serious social problems. Moreover, as a second-hand data source was used with only quantitative information available about attitudes towards radiation-based health concerns, it was not possible to explore the associations between other radiation-related concerns and happiness. Another limitation was that it was not possible to conduct a longitudinal analysis using cumulative data with the same respondents. Such efforts could contribute to illuminating changes in people’s happiness at different stages of disaster recovery. Further research applying both quantitative and qualitative methods over a longitudinal period is thus needed in order to fully comprehend the influencing mechanism of post-disaster public health impacts on happiness.

## Figures and Tables

**Table 1:** Descriptive statistical results of the original dataset.

Variable	Male		Female		*t*	Chi-Square	*p*
	N (%)	Mean (SD)	N (%)	Mean (SD)			
Happiness (1–4)	744 (52.8)	2.16 (0.70)	666 (47.2)	2.05 (0.63)	3.15		<0.01
Mental health (6–24)	720 (52.7)	17.27 (4.08)	647 (47.3)	17.05 (4.16)	0.96		0.34
Physical health (1–5)	739 (52.7)	2.81 (0.96)	664 (47.3)	2.82 (0.96)	−0.36		0.72
Radiation-based health concern (1–4)	728 (52.7)	1.98 (0.89)	653 (47.3)	1.97 (0.83)	0.33		0.74
Property loss (in units of 10,000 JPY)	713 (53.6)	162.60 (682.47)	617 (46.4)	81.35 (337.68)	2.81		<0.01
Feelings of recovery achievement (1–4)	736 (53.0)	3.39 (0.75)	652 (47.0)	3.43 (0.69)	−1.01		0.31
Neighborhood connectedness (2–8)	733 (52.8)	5.46 (1.68)	656 (47.2)	5.22 (1.80)	2.45		0.01
Age (20–93 years)	715 (52.1)	57.33 (16.96)	657 (47.9)	53.67 (16.08)	4.09		<0.01
Education						15.72	<0.01
Vocational school or below	448 (62.5)		465 (72.3)				
College or above	266 (37.1)		174 (27.1)				
Other schooling	3 (0.4)		4 (0.6)				
Marital status						41.42	<0.01
Married	541 (73.3)		420 (63.9)				
Single	131 (17.8)		98 (14.9)				
Divorced or widowed	66 (8.9)		139 (21.2)				
FAI (in units of 10 million JPY)						11.74	0.02
<150	47 (7.8)		55 (10.4)				
150–450	269 (44.6)		221 (41.8)				
450–650	111 (18.4)		108 (20.4)				
650–1000	107 (17.7)		110 (20.8)				
>1000	69 (11.5)		35 (6.6)				
Casualty experience							
I was injured						0.54	0.61
Yes	4 (0.5)		5 (0.8)				
No	739 (99.5)		659 (99.2)				
FaRe injury						0.01	0.92
Yes	14 (1.9)		13 (2.0)				
No	729 (98.1)		651 (98.0)				
FaRe died						0.06	0.81
Yes	24 (3.2)		23 (3.5)				
No	719 (96.8)		641 (96.5)				
FrAc injury						0.00	0.94
Yes	23 (3.1)		21 (3.2)				
No	720 (96.9)		643 (96.8)				
FrAc died						1.46	0.23
Yes	42 (5.7)		48 (7.2)				
No	701 (94.3)		616 (92.8)				

Note: SD—standard deviation, FAI—family annual income, FaRe—family or relatives, FrAc—friends or acquaintances.

**Table 2:** Linear regression results in happiness and independent variables (N = 1232).

Independent Variable	Unstandardized Coefficients		Standardized Coefficients	*t*	Sig.	95% CI for *b*	
	*b*	S.E.	B			Lower	Upper
Physical health	0.07	0.02	0.10	3.60	<0.01	0.03	0.10
Mental health	−0.06	<0.01	−0.39	−14.60	<0.01	−0.07	−0.05
Neighborhood connectedness	0.05	0.01	0.13	4.65	<0.01	0.03	0.07
Age	<0.01	<0.01	0.13	4.30	<0.01	<0.01	0.01
Gender (female as reference)							
Male	0.09	0.03	0.07	2.64	0.01	0.02	0.15
Marital status (married as reference)							
Single	0.24	0.05	0.14	4.92	<0.01	0.14	0.33
Divorced or widowed	0.14	0.05	0.08	2.97	<0.01	0.05	0.24
Education (other schooling as reference)							
College or above	−0.10	0.04	−0.07	−2.89	<0.01	−0.17	−0.03
FAI (over 10 million JPY as reference)							
6.5–10	−0.10	0.04	−0.06	−2.23	0.03	−0.18	−0.01
_cons	2.38	0.14		17.12	<0.01	2.11	2.66
R	0.53						
R2	0.28						
Adjusted R2	0.27						
S.E. for the Estimate	0.55						
F	52.55						
Sig.	<0.01						

Note: S.E. = standard error.

## Appendix A. Survey Questionnaires

**Table:**

Variables	Questions	Values	Degrees
Happiness	In general, do you feel happy?	1 to 4	happy to unhappy
Feelings of recovery achievement	Four years after the Tohoku Earthquake Tsunami and Fukushima accident, how far do you think the goals of recovery in Fukushima Prefecture have been achieved?	1 to 4	good to bad
Radiation-based health concerns	Do you agree that negative health effects caused by nuclear radiation due to the Fukushima accident will emerge in the future?	1 to 4	agree to disagree
Physical health	Overall, how would you describe the current condition of your health?	1 to 5	good to bad
Mental health	Sum of answer scores (1)–(6)		
	(1) I feel mentally burned out	1 to 4	agree to disagree
	(2) I do not feel motivated to work or undertake household chores	1 to 4	agree to disagree
	(3) I feel restless	1 to 4	agree to disagree
	(4) I cannot sleep well	1 to 4	agree to disagree
	(5) I feel depressed. No matter what happens, I feel sick	1 to 4	agree to disagree
	(6) I feel too exhausted to do anything	1 to 4	agree to disagree
Casualty experience			
I was injured	(1) I was injured	Yes or No	
FaRe injury	(2) Family or relatives were injured	Yes or No	
FaRe died	(3) Family or relatives died	Yes or No	
FrAc injury	(4) Friends or acquaintances were injured	Yes or No	
FrAc died	(5) Friends or acquaintances died	Yes or No	
Property loss (in units of 10,000 JPY)	How much damage was there to your property, such as your house or household goods?	Open-ended question	
Neighborhood connectedness	Sum of answer scores (1)–(2)		
	(1) How often do you talk with your neighbors?	1 to 4	often to never
	(2) How often do you tell your neighbors about your troubles, or vice versa?	1 to 4	often to never
Age	Open-ended question		
Gender	Male, female		
Marital status	Married, single, divorced or widowed		
Education	Elementary school, middle school, high school, vocational school, college, undergraduate school and above, other schooling		
Family annual income (in units of 10,000 JPY)	Zero, under 70, 70-150, 150-250, 250-350, 350-450, 450-550, 550-650, 650-750, 750-850, 850-1000, 1000-1200, 1200-1400, 1400-1600, over 1600		

## Appendix B. Response Characteristics (Pooled; N = 1439)

**Table:**

Variable	Male		Female		*t*	Chi-Square	*p*
	N	Mean	N	Mean			
Happiness	760	2.17	679	2.06	3.03		<0.01
Mental health	760	17.23	679	17.03	0.89		0.38
Physical health	760	2.81	679	2.83	−0.50		0.62
Radiation-based health concerns	760	1.99	679	1.98	0.28		0.78
Property loss (in units of 10,000 JPY)	760	179.77	679	113.09	2.26		0.02
Feelings of recovery achievement	760	3.38	679	3.41	−0.85		0.40
Neighborhood connectedness	760	5.46	679	5.22	2.46		0.01
Age	760	57.32	679	53.76	4.04		<0.01
Education							
Vocational school or below		471.6		491.8		17.39	<0.01
College or above		276		178.8			
Other schooling		12.4		8.4			
Marital status							
Married		555.4		430		44.31	<0.01
Single		135.2		102.2			
Divorced or widowed		69.4		146.8			
FAI (in units of 10,000 JPY)							
<150		66		77.8		11.75	0.02
150–450		336.6		290.2			
450–650		141		132.4			
650–1000		130.2		131.6			
>1000		86.2		47			
Casualty experience							
I was injured							
Yes	4		5.2				
No	756		673.8				
FaRe injury							
Yes	14		13				
No	746		666				
FaRe died							
Yes	26		23.4				
No	734		655.6				
FrAc injury							
Yes	24.6		22.6				
No	735.4		656.4				
FrAc died							
Yes	43.6		49.4				
No	716.4		629.6				

## Appendix C. Linear Regression Results (Pooled; N = 1439)

**Table:**

Independent Variable	Fraction Missing Info.	Relative Increase Variance	Relative Efficiency	Unstandardized Coefficients		*t*	Sig.	95% CI for *b*	
				*b*	S.E.			Lower	Upper
Physical health	0.05	0.06	0.99	0.07	0.02	4.01	<0.01	0.04	0.11
Mental health	0.08	0.09	0.98	−0.06	<0.01	−14.28	<0.01	−0.07	−0.05
Neighborhood connectedness	0.04	0.04	0.99	0.05	0.01	4.62	<0.01	0.03	0.07
Age	0.12	0.13	0.98	<0.01	<0.01	4.14	<0.01	<0.01	0.01
Gender (female as reference)									
Male	0.01	0.01	1.00	0.12	0.03	3.67	<0.01	0.06	0.18
Marital status (married as reference)									
Single	0.05	0.05	0.99	0.27	0.05	5.67	<0.01	0.18	0.36
Divorced or widowed	0.01	0.01	1.00	0.17	0.05	3.64	<0.01	0.08	0.26
Education (other schooling as reference)									
College or above	0.02	0.02	1.00	−0.11	0.04	−3.26	<0.01	−0.18	−0.04
_cons	0.06	0.06	0.99	2.33	0.14	16.68	<0.01	2.05	2.60
